# Regulatory Effect of Cinnamaldehyde on Monocyte/Macrophage-Mediated Inflammatory Responses

**DOI:** 10.1155/2010/529359

**Published:** 2010-05-11

**Authors:** Byung Hun Kim, Yong Gyu Lee, Jaehwi Lee, Joo Young Lee, Jae Youl Cho

**Affiliations:** ^1^School of Bioscience and Biotechnology, Institute of Bioscience and Biotechnology, Kangwon National University, Chuncheon 200-701, South Korea; ^2^College of Pharmacy, Chung-Ang University, Seoul 156-756, South Korea; ^3^Department of Life Sciences and Research Center for Biomedical Nanotechnology, Gwangju Institute of Science and Technology, Gwangju 500-712, South Korea

## Abstract

Cinnamaldehyde (CA) has been known to exhibit anti-inflammatory and anticancer effects. Although numerous pharmacological effects have been demonstrated, regulatory effect of CA on the functional activation of monocytes and macrophages has not been fully elucidated yet. To evaluate its monocyte/macrophage-mediated immune responses, macrophages activated by lipopolysaccharide (LPS), and monocytes treated with proaggregative antibodies, and extracellular matrix protein fibronectin were employed. CA was able to suppress both the production of nitric oxide (NO) and upregulation of surface levels of costimulatory molecules (CD80 and CD69) and pattern recognition receptors (toll-like receptor 2 (TLR2) and complement receptor (CR3)). In addition, CA also blocked cell-cell adhesion induced by the activation of CD29 and CD43 but not cell-fibronectin adhesion. Immunoblotting analysis suggested that CA inhibition was due to the inhibition of phosphoinositide-3-kinase (PI3K) and phosphoinositide-dependent kinase (PDK)1 as well as nuclear factor-(NF-) *κ*B activation. In particular, thiol compounds with sulphydryl group, L-cysteine and dithiothreitol (DTT), strongly abrogated CA-mediated NO production and NF-*κ*B activation. Therefore, our results suggest that CA can act as a strong regulator of monocyte/macrophage-mediated immune responses by thiolation of target cysteine residues in PI3K or PDK1.

## 1. Introduction

Monocytes/macrophages play a critical role in managing innate and adaptive immunity-including inflammatory processes by secreting proinflammatory molecules (eg. tumor necrosis factor (TNF)-*α*, and nitric oxide (NO)) [1]. The activation of macrophages and monocytes is mediated by activation of various receptors including Toll like receptor-(TLR-) 4 and their counter molecules such as lipopolysaccharide (LPS) derived from bacteria or virus [2]. In parallel, the activation of these cells triggers various cellular responses such as cell migration, adhesion, extravasation, and infiltration to induce effective movement of these cells into inflamed tissue by adhesion molecules such as *β*1 (CD18) or *β*2 (CD29) integrins and their ligands such as vascular cell adhesion molecule-(VCAM-) 1 or intercellular adhesion molecule-(ICAM-)1 [3]. The molecular interaction between surface receptors and counter molecules seen in various cellular inflammatory responses generates a series of complex signaling events composed of *numerous intracellular enzymes* such as phosphoinositide-3-kinase (PI3K), phosphoinositide-dependent kinase 1 (PDK1), Akt (protein kinase B), and mitogen-activated protein kinases (MAPKs) such as extracellular signal-regulated kinase (ERK), c-Jun N-terminal kinase (JNK), and p38 [4, 5] linked to actin cytoskeleton rearrangement for modulating cellular activation or the proinflammatory gene expression by mediating with transcription factors like NF-*κ*B and AP-1 [6]. Recently, inflammatory responses by monocytes and macrophages were reported to be a critical pathological event in triggering various acute or chronic diseases such as septic shock, cancer, autoimmune diseases, cardiovascular diseases, obesity, and diabetes [7, 8]. It is therefore considered that development of promising regulators of monocyte/macrophage-mediated inflammatory responses without side effects could be useful for prevention of, or as the therapeutic remedy for, various inflammation-mediated diseases [9].

**Figure 1:**
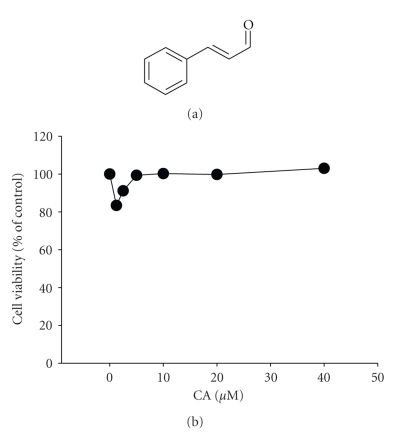
Effect of CA on the viability of RAW264.7 cells. (a) Chemical structure of CA. (b) The viability of RAW264.7 cells was determined under the same conditions by MTT assay after 24 hour incubation.

Cinnamaldehyde (CA; Figure 1(a)), a major bioactive compound isolated from the leaves of *Cinnamomum osmophloeum* Kaneh [10, 11], has been known to trigger apoptosis through mitochondrial permeability transition in human promyelocytic leukemia HL-60 cells [12], by activating the proapoptotic Bcl-2 family proteins [13]. Treatment of cultured mouse splenocytes with CA in a dose-dependent manner blocked the proliferation of lymphocytes induced by concanavalin A and LPS [14]. This compound was also found to suppress NF-*κ*B activation within macrophage-like RAW264.7 cells [15]. It has been demonstrated that CA is capable of blocking inducible nitric oxide synthase (iNOS) and NO production by mediation of NF-*κ*B activation blockade in LPS-stimulated RAW264.7 cells [16]. Moreover, the production of PGE_2_ was also reduced by CA exposure in cultured rat cerebral microvascular endothelial cells [17]. These results strongly *suggested* that CA can be applied as an anti-inflammatory drug. However, the pharmacological target and inhibitory mechanism of CA, and its activity on various cellular events such as cell adhesion and migration commonly seen in the functional activation of monocytes/macrophages, have not been examined yet. Thus, in this study, we investigated the detailed regulatory roles of CA on monocyte/macrophage-mediated immune responses and its potential target enzyme.

## 2. Materials and Methods

### 2.1. Materials

CA was kindly supplied from the Aging Tissue Bank (Pusan National University, Busan, South Korea). LPS, phorbol 12-myristate 13-acetate (PMA), FITC-dextran, 1,4-dithiolthreitol (DTT), L-cysteine, and TNF-*α* were obtained from Sigma Chemical Co. (St. Louis, MO). LY294002 and wortmannin and U0126 were from Calbiochem (La Jolla, CA). RAW264.7 and TLR4-expressing HEK293 cells were purchased from American Type Culture Collection (Rockville, MD) and Invivogen (San Diego, CA). All other chemicals were purchased from Sigma. Fibronectin was obtained from BD Biosciences (San Diego, CA). Phospho-specific antibodies to p85, PDK1, Akt, and *I* *κ* *B* *α*  were obtained from Cell Signaling (Beverly, MA). Cell-cell adhesion-inducing antibodies to CD29 (MEM 101A, purified IgG1), CD43 (161-46, ascites, IgG1), and P5D2 were used as reported previously [18, 19]. Antibodies to costimulatory (CD80, CD86, CD40, and CD69) and adhesion (CD29, CD43, and CD18) molecules were from BD Biosciences (San Diego, CA). Antibodies to pattern recognition receptors (dectin-1, TLR2, TLR4, SR, and CR3) were purchased from Serotec (Raleigh, NC). Suntide, a peptide sequence derived from Akt (protein kinase B) [20], was synthesized *by* Peptron (Daejeon, South Korea).

### 2.2. Cell Culture

RAW264.7 and TLR4-expressing HEK293 cells were cultured in RPMI1640 medium supplemented with 10% heat-inactivated fetal bovine serum (Gibco, Grand Island, NY), glutamine, and antibiotics (penicillin and streptomycin), at 37°C with 5%  CO_2_.

### 2.3. NO Production

The inhibitory effect of CA on NO production was determined as previously described [21]. CA solubilized with DMSO (100%) was diluted with RPMI1640. RAW264.7 cells (2 × 10^6^ cells/ml) were incubated with LPS (1 *μ*g/ml) in the presence or absence of CA for 24 h. Supernatants were assayed for NO and TNF-*α* contents using Griess reagent.

### 2.4. Luciferase Reporter Gene Activity Assay

Since RAW264.7 cells are not easily transfected with certain types of DNA constructs, TLR4-expressing HEK293 cells (1 × 10^6^ cells/ml) were used to be transfected with 1 *μ*g of plasmid with NF-*κ*B and *β*-galactosidase by using the calcium phosphate method in a 12-well plate. The cells were used for experiments 48 h after transfection. Luciferase assays were performed using the Luciferase Assay System (Promega) [22].

### 2.5. Determination of Phagocytic Uptake

To measure the phagocytic activity of RAW264.7 cells, a previously reported method was used with slight modifications [23]. RAW264.7 (5 × 10^4^) cells pretreated with CA were resuspended in 100 *μ*l PBS containing 1% human serum and incubated with FITC-dextran (1 mg/ml) at 37°C and 0°C for 30 min. The incubation was stopped by the addition of 2 ml ice-cold PBS containing 1% human serum and 0.02% sodium azide. The cells were washed three times with cold PBS-azide and analyzed by flow cytometry.

### 2.6. Cell-Cell or Cell-Extracellular Matrix Protein (Fibronectin) Adhesion Assay

U937 cell adhesion assay was performed as previously reported [24, 25]. Briefly, U937 cells maintained in complete RPMI1640 medium (supplemented with 100 U/ml of penicillin 100 *μ*g/ml of streptomycin, and 10% FBS) were preincubated with CA for 1 h at 37°C and further incubated with aggregation-inducing (agonistic) antibodies (1  *μ*g/ml) in a 96-well plate. After a 50 minute incubation, cell-cell clusters were determined by homotypic cell-cell adhesion assay using a hemocytometer [24] and analyzed with an inverted light microscope equipped with a COHU high-performance CCD (Diavert) video camera. For the cell-fibronectin adhesion assay, CA-treated U937 cells (5 × 10^5^ cells/well) were seeded on a fibronectin-coated (50 *μ*g/ml) plate and incubated for 3 h [26]. After removing unbound cells with PBS, the attached cells were treated with 0.1% crystal violet for 15 min. The OD value at 540 nm was measured by a Spectramax 250 microplate reader.

### 2.7. Cell Migration (Wound Healing) Assay

RAW264.7 cells (2 × 10^6^ cells/ml) were incubated with CA for 30 min. After scratching the cultured cells with a pipette, the cells were further incubated for 48 h. The images of the cells in culture were obtained using an inverted phase contrast microscope attached to a video camera.

### 2.8. Flow Cytometry

Surface levels of adhesion molecules (CD29, CD43, and CD18) in U937 and co-stimulatory molecules (CD69, CD80, CD40, and CD86) and pattern recognition receptors (dectin-1, TLR2, TLR4, SR, and CR3) in RAW264.7 cells were determined by flow cytometric analysis as reported previously [24]. Stained cells were analyzed on a FACScan device (Becton-Dickinson, San Jose, CA).

### 2.9. MTT Assay

Cell proliferation was measured by 3-(4,5-dimethylthiazol-2-yl)-2,5-diphenyltetrazolium bromide (MTT) assay as described previously [27].

### 2.10. Extraction of Total RNA and Semiquantitative RT-PCR Amplification

The total RNA from the CA and LPS-treated RAW264.7 cells was prepared by adding TRIzol Reagent (Gibco BRL), according to manufacturer's protocol. Semiquantitative RT reactions were conducted using MuLV reverse transcriptase as reported previously [28]. The primers (Bioneer, Daejeon, South Korea) were used as previously reported [29].

### 2.11. Preparation of Cell Lysates and Immunoblotting

RAW264.7 cells (5 × 10^6^ cells/ml) were washed 3 times in cold PBS with 1 mM sodium orthovanadate and lysed in lysis buffer (20 mM Tris-HCl, pH 7.4, 2 mM EDTA, 2 mM ethyleneglycotetraacetic acid, 50 mM *β*-glycerophosphate, 1 mM sodium orthovanadate, 1 mM dithiothreitol, 1% Triton X-100, 10% glycerol, 10 *μ*g/ml aprotinin, 10 *μ*g/ml pepstatin, 1 mM benzimide, and 2 mM PMSF) for 30 min with rotation at 4°C. The lysates were clarified by centrifugation at 16,000 × g for 10 min at 4°C and stored at −20°C until needed. Whole cell lysates were then analyzed by immunoblotting. Proteins were separated on 10% SDS-polyacrylamide gels and transferred by electroblotting to polyvinylidene difluoride (PVDF) membrane. Membranes were blocked for 60 min in Tris-buffered saline containing 3% bovine serum albumin, 20 mM NaF, 2 mM EDTA, and 0.2% Tween 20 at room temperature. The membrane was incubated for 60 min with specific primary antibody at 4°C, washed 3 times with the same buffer, and incubated for additional 60 min with horse radish peroxidase-(HRP-) conjugated secondary antibody. The total and phosphorylated levels of p85, PDK1, Akt, I*κ*B*α*, and *β*-actin were visualized using the ECL system (Amersham, Little Chalfont, Buckinghamshire, UK).

### 2.12. Matrix-Assisted Laser Desorption Ionization Time-of-Flight Mass Spectrometry (MALDITOF/MS)

*α* Cyano-4-hydroxycinnamic acid (20 mg) (Bruker Daltonics, Bremen, Germany) was dissolved in 1 ml acetone  :  ethanol (1  :  2, v/v), and 0.5 *μ*l of the matrix solution was mixed with an equivalent volume of sample. Analysis was performed using an Ultraflex TOF/TOF system (Bruker Daltonics). The Ultraflex TOF/TOF system was operated in positive ion reflect mode. Each spectrum was the cumulative average of 250–450 laser shots. Mass spectra were first calibrated in the closed external mode using the peptide calibration standard II (Bruker Daltonics), sometimes using the internal statistical mode to achieve maximum calibration mass accuracy.

### 2.13. Statistical Analysis

The Student's *t*-test and one–way ANOVA were used to determine the statistical significance between values of the various experimental and control groups. *P* values of  .05 or less were considered to be statistically significant.

## 3. Results and Discussion

Monocytes/macrophages are the prime immune cells managing inflammatory responses, which *contribute to development of number of diseases such as cancer, diabetes, and atherosclerosis* [30, 31]. This view led us to develop novel immunoregulatory drugs based on the functional activation of monocytes and macrophages without side effects to prevent such diseases. In this context, medicinal plants that have traditionally been used for long time are considered as attractive biopharmaceutical candidates. With this goal, therefore, we have attempted to develop macrophage function regulators using naturally occurring compounds or plants for a decade. 

**Figure 2:**
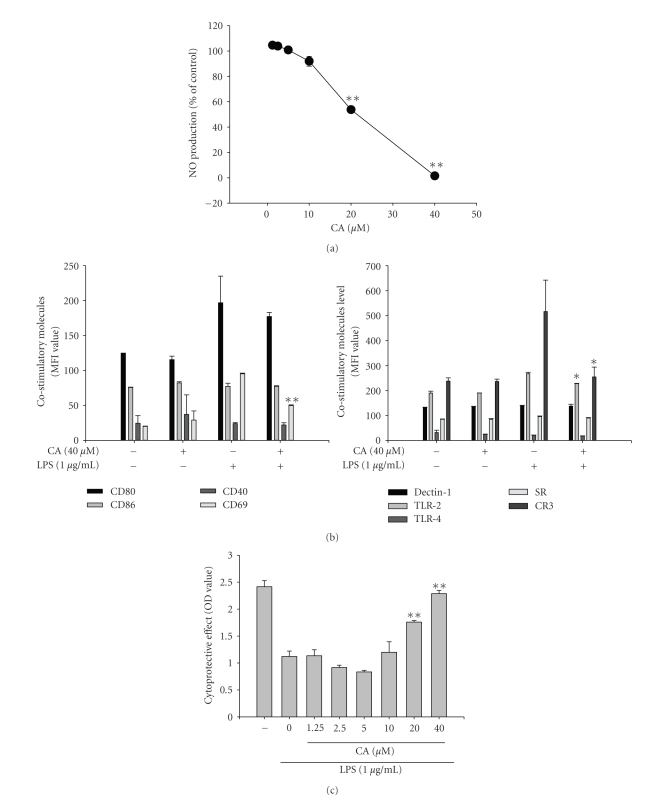
Effect of CA on the production of NO and surface upregulation of costimulatory molecules and pattern recognition receptors in LPS-activated RAW264.7 cells. RAW264.7 cells (2 × 10^6^ cells/ml) were incubated with concentrations of CA in the presence of LPS (1 *μ*g/ml) for 24 h (NO), 12 h (costimulatory molecules and pattern recognition receptors), or 24 h (cytoprotective effect). NO levels (a) in culture supernatant were determined by Griess assay. Surface levels of CD40, CD69, CD80, CD86, dectin-1, TLR2, TLR4, SR, and CR3 were determined by flow cytometric analysis. The viability of RAW264.7 cells was determined by MTT assay. *P < .05 and **P < .01 represent significant difference compared to LPS alone.

**Figure 3:**
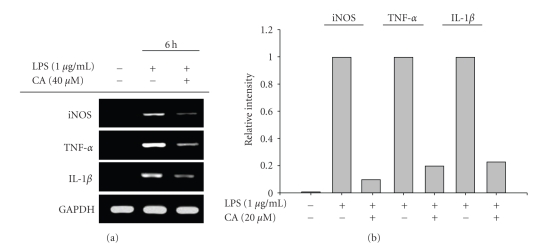
Effect of CA on mRNA levels of inflammatory genes in LPS-activated RAW264.7 cells. (a and b) RAW264.7 cells (5 × 10^6^ cells/ml) were incubated with CA in the presence of LPS (1 *μ*g/ml) for 6 h. (a) The mRNA levels of TNF-*α*, iNOS, IL-1*β*, and GAPDH were determined by RT-PCR. (b) Relative intensity was determined by densitometric scanning.

**Figure 4:**
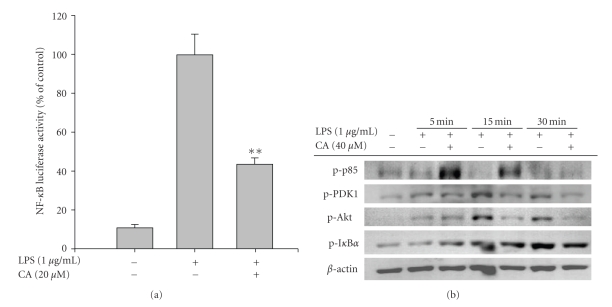
Effect of CA on the upstream signaling pathway for transcriptional activation of NF-*κ*B. (a) TLR-4-expressing HEK293 cells co-transfected with the plasmid construct, NF-*κ*B-Luc (1 *κ*g/ml), and *β*-gal (as a transfection control) were treated with CA in the presence or absence of LPS (1 *μ*g/ml) for 18 h, and luciferase activity was determined by luminometry. Data represents mean ± SEM of three independent observations performed in triplicate. (b) RAW264.7 cells (5 × 10^6^ cells/ml) pretreated with CA for 1 h were stimulated with LPS (1 *κ*g/ml) for indicated times. After immunoblotting, the levels of phosphorylated forms of p85, PDK1, Akt, and I*κ*B*α* were identified by corresponding antibodies. The data presented here is from one experiment, representative of three done in total. **P < .01 represents significant difference compared to LPS alone.

**Figure 5:**
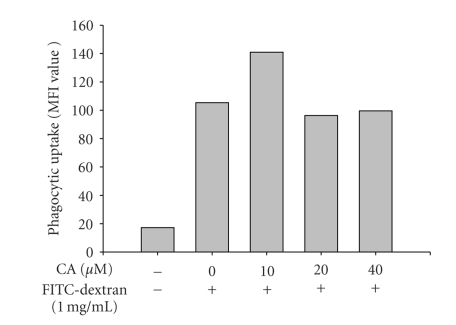
Effect of CA on the phagocytic uptake of FITC-labeled dextran. RAW264.7 cells (1 × 10^6^) were incubated with CA in the presence or absence of 1 mg/ml of FITC-labeled dextran for 30 min. The uptake level of dextran was determined by flow cytometric analysis.

The regulatory effect of CA on LPS-induced macrophage immune responses was initially examined. Upon nontoxic concentrations (0 to 40 *μ*M) (Figure 1(b)), CA strongly suppressed the production of NO (Figure 2(a)) and the surface upregulation of costimulatory (CD80 and CD69) and pattern recognition (TLR2 and CR3) molecules (Figure 2(b)). Moreover, CA protected cells from LPS-induced cytotoxicity and apoptosis, mainly induced by the NO produced (Figure 2(c)). The inhibition of NO release occurred at the transcriptional levels, according to Figure 3. Thus, CA blocked mRNA expression of iNOS as well as other proinflammatory cytokines such as TNF-*α* and IL-1*β* as much as 80 to 95% (Figure 3). Because transcriptional downregulation of inflammatory mediators by CA has been reported to inhibit NF-*κ*B activation [16, 32], reporter gene assay for NF-*κ*B and immunoblotting analysis of upstream signaling were further conducted. As Figure 4(a) shows, CA blocked NF-*κ*B-mediated luciferase activity induced by LPS treatment, similar to previous papers [15, 32]. Interestingly, CA also blocked a series of NF-*κ*B activation signaling pathways. This compound suppressed the phosphorylation of I*κ*B*α*, Akt, and PDK1 but not p85, a regulatory subunit of PI3K (Figure 4(b)), suggesting that the pharmacological target of CA may be PI3K or PDK1 in LPS-mediated macrophage immune responses. Unlike LPS-induced inflammatory responses, FITC-dextran-induced phagocytic uptake of RAW264.7 cells, a major response found in innate immunity, was not negatively modulated by this compound (Figure 5), which suggests that CA cannot regulate all macrophage functions but modulates mostly LPS-mediated immune responses in macrophages. 

**Figure 6:**
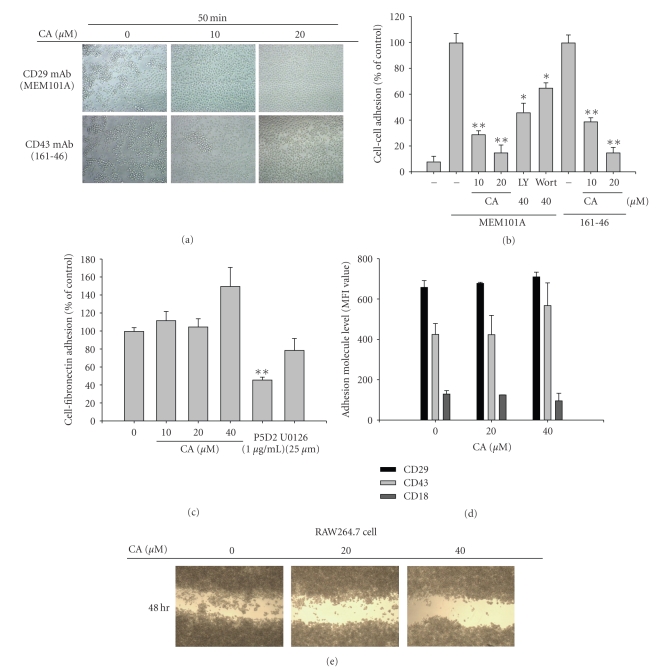
Effect of CA on cell-cell adhesion and cell-fibronectin adhesion. U937 cells pretreated with CA for 1 h were incubated in the presence or absence of anti-CD29 antibody MEM101A (1 *μ*g/ml) or anti-CD43 antibody 161-46 (1 *μ*g/ml). Images (a) of the cells in culture were obtained using an inverted phase contrast microscope attached to a video camera. Quantitative analysis (b) of cell-cell clusters was assessed using a quantitative cell-cell adhesion assay. (c) U937 cells pretreated with CA, P5D2, or U0126 for 30 min were seeded on fibronectin-coated (50 *μ*g/ml) plates and further incubated for 3 h. The number of attached cells was determined by crystal violet assay. (d) U937 cells (1 × 10^6^cells/ml) were incubated with CA for 12 h. Surface levels of CD29, CD18, and CD43 were determined by flow cytometric analysis. (e) RAW264.7 cells (2 × 10^6^ cells/ml) pretreated with CA for 1 h were incubated for 48 h after scratching with a pipette. Images of the cells in culture were obtained using an inverted phase contrast microscope attached to a video camera.   *P < .05 and **P < .01 represent significant difference compared to control.

Meanwhile, CA also strongly suppressed cell-cell adhesions induced by proaggregative antibodies to CD29 and CD43 up to 85% (Figures 6(a) and 6(b)). However, adhesion of U937 cells to fibronectin, an extracellular matrix protein acting as a CD29 ligand, was not suppressed by this compound (Figure 6(c)), indicating that adhesion between cells but not cell and extracellular matrix could be blocked by CA. However, CA did not suppress the surface levels of adhesion molecules such as CD18, CD29, and CD43 even at 40 *μ*M (Figure 6(d)), while CA diminished the migration of RAW264.7 cells in an in vitro wound healing assay, compared to normal (Figure 6(e)). Considering that cell-fibronectin adhesion only requires simple activation of CD29, and while intracellular signaling (ERK, p38, and protein kinase C*δ*) and actin cytoskeleton change are important factors in cell-cell adhesion events [24, 25], CA seems to modulate intracellular signaling events rather than the blockade of a direct interaction between CD29 and fibronectin. In particular, these signaling events targeted to CA seem to be involved in modulating cell migration commonly seen in both cell-cell adhesion (Figure 6(a)) and wound healing assays (Figure 6(e)). CD29-mediated cell-cell adhesion is an essential phenomenon for survival and activation of immune cells, particular for an interaction between antigeny presenting cells (APC) and T lymphocytes or NK cells. Therefore, antiaggregative effect of CA may contribute to the regulation of monocyte/macrophage roles as APC requiring their adhesion responses. Similarly, since PI3K/Akt inhibition by LY294002 and wortmannin suppressed CD29-mediated cell-cell adhesion, CA inhibition of adhesion and migration events seems to occur at the level of PI3K/PDK1, as in the case of LPS signaling. 

**Figure 7:**
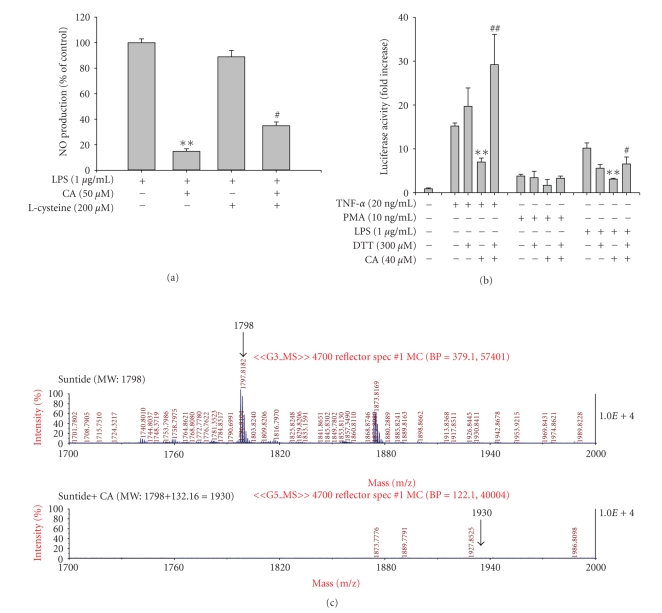
Effect of thiol compounds on CA inhibition of NO production and NF-*κ*B-mediated luciferase activity. (a) RAW264.7 cells (2 × 10^6^ cells/ml) pretreated with L-cysteine for 30 min were incubated with indicated concentrations of CA in the presence of LPS (1 *μ*g/ml) for 24 hours. NO levels in culture supernatant were determined by Griess assay. (b) TLR-4-expressing HEK293 cells cotransfected with the plasmid construct, NF-*μ*B-Luc (1 *μ*g/ml), and *β*-gal (as a transfection control) were treated with CA, L-cysteine or DTT in the presence or absence of TNF-*μ*, PMA, or LPS for 18 h where luciferase activity was determined by luminometry. (c) CA and Suntide were incubated in kinase assay buffer for 30 min. The adduct was then identified by MALDITOF/MS analysis. **P < .01 and ^#^ *P* < .05 represent significant difference compared to control or CA groups.

Since thiol compounds such as DTT and L-cysteine are reported to block CA inhibition [16], we finally examined whether this pattern can be observed under the same conditions. As expected, pretreatment of L-cysteine (200 *μ*M) or DTT (300 *μ*M) before CA treatment abrogated the inhibitory activity of CA in both NO production (Figure 7(a)) and NF-*κ*B-mediated luciferase activity (Figure 7(b)), suggesting that thiolation is the major chemical mechanism of CA inhibition. Up to date, we have not been able to locate the exact thiolation site on the target protein by CA. However, recent findings revealing that an adduct formation of hydroquinone with the sulfhydryl group of Cys-310 in Akt is able to block the phosphorylation of both Thr-308 and Ser-473 [33] seem to suggest that a target cysteine sequence of PI3K or PDK1 can serve as a thiolation site affecting their phosphorylation and activation. Indeed, we failed to detect an adduct formation between CA and Suntide, a peptide fragment containing Cysteine-310 designed according to Akt amino acid sequence (Figure 7(c)). Because identification of CA target thiolation site is an important step to understand the exact molecular mechanism of CA inhibition, we are currently undertaking further analysis using other peptide sequences containing cysteine residues from PI3K and PDK1.

In conclusion, we found that CA was able to suppress the production of NO and upregulation of surface levels of costimulatory molecules such as the surface upregulation of both costimulatory (CD80 and CD69) and pattern recognition molecules (TLR2 and CR3). In addition, CA also blocked both cell migration and cell-cell adhesion induced by CD29 and CD43, but not cell-fibronectin adhesion. The CA inhibition was likely due to the inhibition of PI3K and PDK1, important for NF-*κ*B activation of signaling components, according to immunoblotting analysis. In particular, L-cysteine and DTT strongly *interfered CA-mediated inhibition* of NO production and NF-*κ*B activation. Therefore, our results suggest that CA can act as a strong regulator of monocyte/macrophage-mediated immune responses, possibly by the induction of thiolation at cysteine residues in the target enzyme (PDK1 or PI3K). To prove a detailed inhibitory mechanism, identification of molecular targets of CA will be investigated in our next series of experiments.
